# Hair transparency decoding in Asia: From stylists’ perception to in vitro measurement

**DOI:** 10.1111/srt.13083

**Published:** 2021-12-05

**Authors:** Lu Zheng, Noriko Matsumoto, Anthony Galliano, Shuchismita Basu, Karl Wunsch, Olivia Isard, Chikayo Nakao, Alexandre Nicolas, Damien Velleman

**Affiliations:** ^1^ Nihon L’OREAL K.K Research & Innovation Kawasaki Japan; ^2^ L'Oréal Research and Innovation Saint‐Ouen France; ^3^ Present address: Nihon L'OREAL K.K. R&I, KSP Kanagawa‐ken Japan

**Keywords:** consumer centric, digital, hair transparency, instrumental evaluation, trendy hair color

## Abstract

**Background:**

The concept of hair transparency has been claimed widely in the Japan (and now it is spreading to Asian) hair color market. Despite the general use of this concept, to date, there is no clear and objective description to accurately explain what it is. In this work, we have decoded and gave clarity to the concept of hair transparency via a technical model (validated for both Japan and China markets) composed of measurable parameters of hair property using a single device.

**Methodology and Results:**

A comprehensive study composed of various tests was used, starting with a qualitative identification of key parameters via in‐depth workshop discussions with over 40 Japanese stylists and a panel of 12 consumers. These identified parameters (luminosity, color visibility, and Shine) were then translated into technically measurable parameters of the hair fiber (Diffused light intensity, ratio of RGB channel intensities of Diffused light, and luster) via a single instrument—Hair SAMBA (a dual‐polarized imaging system). Afterward, 10 carefully selected anchor shades were used as visual stimuli in an online pairwise comparison (PC) study with 100 Japanese stylists to generate quantitative transparency perception data of the swatches. Technical parameters of these swatches were measured by SAMBA and consolidated with the PC output, for the creation and validation of the mathematical model. After, with another PC study (N = 100) in China, with seven shades from Japan study and 6 additional Chinese market shades, the applicability of the model in China market was validated.

**Conclusion:**

We have clarified and quantified the concept of hair transparency through a consumer centric approach and with objective data. Our findings will enable the development of optimum transparent shades which better suits consumer needs. Lastly, we would like to highlight the beauty of digitalization in the study: The digital evaluation pathways chosen allowed us to collect quantitative consumer data from two countries for the creation of a robust model under the impact of COVID‐19 and would definitely be the way to go for our future consumer evaluation studies.

## 1 INTRODUCTION

Since 2018, hair transparency (“Tōmei‐kan” 透明感) has become a fashion buzz word in Japan for trending hair color and is making its way to China and other East Asian countries. From physics’ point of view, transparency is a material property that characterizes its ability to let light pass through. However, the stylists’ and consumers’ perception of hair transparency is rather complex. Transparency has been claimed widely in Japan hair color market, and despite the high penetration, to date, there is no clear and objective description to accurately explain what it is. In this work, we aim to decode and give clarity to the Japanese trend of hair transparency, by quantification of Japanese/Chinese stylists’ perception with a mathematical model, which paves the way for the development of optimum transparent shades to better fulfill consumer needs.

To achieve this goal, a comprehensive study composed of various tests was launched, starting with the qualitative identification of key parameters via in‐depth workshop discussions with over 40 Japanese stylists and a panel of 12 consumers. These identified parameters (eg, color, luminosity, and Shine) were then translated into technically measurable parameters of hair fiber via a single instrument—Hair SAMBA (a dual‐polarized imaging system developed by Bossa Nova Technologies, USA). Afterward, standard images of 10 carefully selected anchor shades covering wide ranges of the identified parameters were used as visual stimuli in a pairwise comparison (PC) study with 100 Japanese stylists to generate quantitative transparency perception data of the swatches. Technical parameters of these swatches were measured by SAMBA and consolidated with the PC output, for the creation of the mathematical model. The same PC study with additional shades was launched with 100 Chinese stylists to validate the applicability of the model to China market.

## 2 METHODOLOGY

To decode hair transparency trend, Nihon L’Oréal Consumer & Market Insight (CMI) has conducted two studies, one with 12 consumers who place high importance on “transparent hair color” for ideal hair color and another with 40 top Japanese stylists who are actively communicating on “hair transparency.” In both studies, it was found that hair transparency has three dimensions: *Color*—high luminosity and light throughness (often achieved through bleaching agents) with neutralized undertone (red or orange/yellow undertone, especially after bleach application, ie, removal of undertone for color sheerness), color visibility (high contrast between hair colors with/without bright (sun)light); *Appearance (Shine*)—healthy with beautiful reflect; and *Texture*—fluidic movement, soft, and fine hair. In this paper, to simplify the message, we will attempt to capture the perception of hair transparency with the more straightforward visual parameters: *color* and *Shine* (Shine is also linked to surface smoothness^1^). The texture (*tactile*) aspect will be covered by a follow‐up study separately.

**FIGURE 1:**
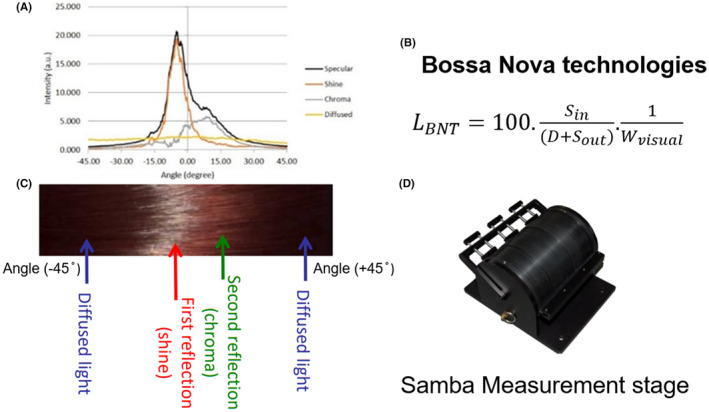
Illustration of SAMBA hair measurement. (A) Representative profiles of Specular, Shine, Chroma, and Diffused bands of Japanese slightly bleached hair (L*=36.1 a*=11.3 b*=21.9). (B) Luster equation by Bossa Nova, where S = specular, D = Diffused, and W = specular bandwidth. (C) Image reconstructed with the measurement profiles. (d) SAMAB hair measurement stage

To numerically describe hair transparency, we will first need to identify a device which allows us to simultaneous measure the integration of these parameters. Various studies have been conducted on hair Shine measurements in the past^2^, ^3^, ^4^ and on understanding human perception of hair Shine.^5^, ^6^, ^7^ SAMBA Hair System^®^ (a dual‐polarized imaging system developed by Bossa Nova Technologies, USA) has been established to measure hair luster (Shine, See *L_BNT_* Eqn. in Figure 1B) and is an industrial standard device for hair Shine measurement.^8^ By using a polarization camera system, after illumination, hair “Shine” (first reflection, no color information), “Chroma” (second reflection inside of hair fiber, with color information), and “Diffused” (scattering inside of hair, with color information) can be measured and represented separately, where the sum of Shine and Chroma profiles gives Specular profile. For each of the three profiles, the intensity profiles of R, G, and B channels are captured by the camera, which contains color information.^8^, ^9^, ^10^ Hair Shine and surface smoothness were successfully measured by SAMBA previously.^1^, ^11^ In a follow‐up study by the same group,^12^ the overlapping degrees between “Shine” and “Chroma” were used in combination with luster values, to compute hair color vibrance factor (*HCVF*). They have found that hair color had an impact on *HCVF*, and the higher the *HCVF* value, the more vibrant the hair color looks. A skin translucency study by Matsubara (P&G)^13^ has demonstrated that the sensorial perception of skin translucency (glow, fairness, and fine texture) could be translated into numerical descriptions using SAMBA face system^®^ (which shares largely similar working principles with the SAMBA hair system^®^) by looking at the specular rate profiles in RGB channels individually. In this study, we aim to develop the first mathematical model in the industry, which describes hair transparency objectively, by simultaneously measurements of color and Shine parameters, using SAMBA hair system^®^.

## 3 RESULTS AND DISCUSSION

### 3.1 Luminosity, color visibility, and Shine measurement with SAMBA

**FIGURE 2:**
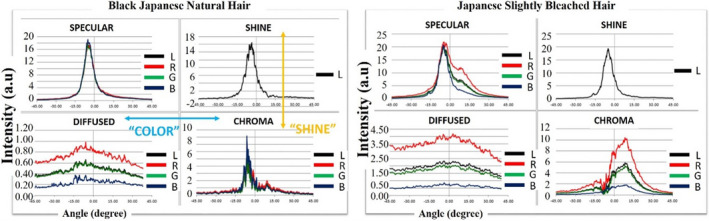
SAMBA measurement profiles of hair specular (Shine and Chroma) and Diffused light intensities in different channels—R, G, B, and L (averaged overall) of black natural hair and slightly bleached hair. Overall, natural black hair has a *narrower specular bandwidth*, lower Diffused light intensity, and *lower red intensity ratio in the Diffused profile*

Since luminosity, color visibility, and Shine are identified as important parameters to describe transparency, we will first look at how they can be measured by SAMBA. Luminosity (brightness) of the hair can be captured by the amount of Diffused light measured in SAMBA, as it is directly related to how much light is absorbed by hair fiber. The higher the hair luminosity, the higher the Diffused light parameter. For color information, as SAMBA camera is able to separately capture information in R, G, and B channels, with the *Diffused* channel has the most intense color information (multiple light‐fiber interaction instead of one time only in *Chroma* channel). The relative intensities of RGB channels can be used to express hair color properties. For Shine, the luster value established by Bossa Nova (*L_BNT_*) is widely used for the quantification of hair Shine in the cosmetic industry. To illustrate how color/Shine information can be interpreted by looking at profiles measured by SAMBA hair, let us take a look at Figure 2. Profile of Japanese black natural hair (L* = 19.8, a*=1.8, b* = 1.7) was shown on the left side and slightly bleach hair (L* = 36.1, a* = 11.3, b* = 21.9) shown on the right side. (i) When hair is darker, the Shine and Chroma bands overlap greatly, which gives narrower specular bandwidth, often leads to higher luster values (in the example here, *L*_BNT_ = 24.5 for black hair and 12.1 for slightly bleached hair) and vice versa (ii) intensity of Diffused band increased with increment of L*, which is consistent with the fact that lighter hair color allows more light to pass through and diffuses more light; and (iii) the proportions of RGB intensities change with a* and b* values of hair, where redder and yellower hair (slightly bleached) has higher R intensity percentage (60%) than black hair (50%) in Diffused profile.

### 3.2 Mathematical description of hair transparency using SAMBA data

Based on our observations and understanding of the established interaction mechanism between hair fiber and incident light, we have defined a new parameter—Hair Transparency Index (HTI) to capture the comprehensive effects of hair Shine and color, to describe consumer/stylist perceived hair transparency as follows:

$$\mathrm{HTI}={\mathrm{Luminosity}}^{\mathrm{color}\;\mathrm{visibility}}+\mathrm{Shine}$$

where $\mathrm{Luminosity}=\;\frac{\log\left({\mathrm{diffused}}_{L_{\mathrm{int}}}\right)}{\log\left(100\right)}$

$$\mathrm{Color}\;\mathrm{visibility}=\;\frac{{\mathrm{diffused}}_{{(R}_{\mathrm{int}}+G_{\mathrm{int}}+B_{\mathrm{int}})}}{{\mathrm{diffused}}_{{(R}_{\mathrm{int}})}}$$

$$\mathrm{Shine}=\frac{L_{\mathrm{BNT}}}{75}$$

All the parameters can be directly obtained from SAMBA hair data file. *L*_BNT_ is the Shine parameter. $\begin{array}{c} {\mathrm{diffused}}_{R_{\mathrm{int}}},\;\mathrm{diffuse}d_{G_{\mathrm{int}}}, \end{array}$ $\begin{array}{c} \;\mathrm{diffuse}d_{B_{\mathrm{int}}},\;\mathrm{and}\;\mathrm{diffuse}d_{L_{\mathrm{int}}} \end{array}$ represent the integral of R, G, B, and L(overall) channel intensities in the obtained Diffused profile respectively. First part of the equation takes care of the color impact on hair transparency definition—(1) higher luminosity in general gives higher HTI (higher luminosity leads to higher perceived transparency by stylists); (2) in terms of color visibility, when hair base color is dark (eg, Asian hair at a no or low bleach level with high melanin content) (correspondingly in SAMBA, we characterize hair is in the dark group when ${\mathrm{diffused}}_{L_{\mathrm{int}}}$ < 100), warmer shades (eg, red and pink) tend to be more visible and perceived to be of higher transparency (ie, in SAMBA, the higher the R (red) channel intensity); and when hair base color is bright (eg, Asian hair at high bleach level with more apparent red/orange undertone) (in SAMBA, ${\mathrm{diffused}}_{L_{\mathrm{int}}}$ > 100), the better coverage of the hair orange/red undertone (in SAMBA, the lower the R (red) channel intensity), the higher the perceived transparency. Note that because of this difference on color impact, the comparison of HTI values of swatches in the dark group and bright group will not be applicable in this model. Second part of the equation considers for Shine; the higher the Shine and healthier hair appears (in SAMBA, higher *L*_BNT_ values), the more transparency perceived. The value 75 at the denominator was used to adjust the weightage of the parameters in determining hair transparency.

### 3.3 Validation of the technical model with quantitative stylists’ perception data in Japan

**FIGURE 3:**
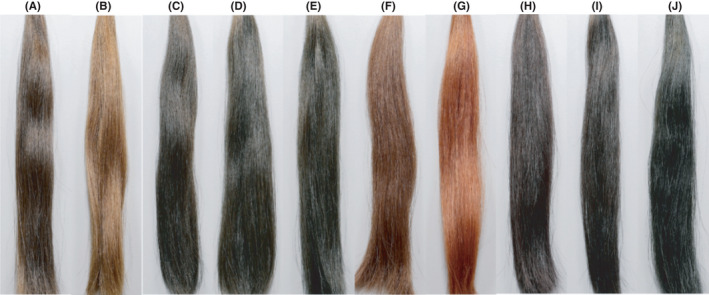
Visual stimuli used in the paired comparison study with 100 Japanese stylists. Bright group: (B), (F), and (G). Dark group: the rest

After defining HTI parameter based on qualitative learnings from stylists and our understanding of SAMBA parameter, to validate our HTI model, swatches colored with 10 carefully selected anchor shades (Figure 3), covering wide ranges of the identified parameters (eg, color, luminosity, and Shine), were prepared and measured with SAMBA to compute their level of transparency. At the same time, standard images of the 10 anchor shades were used as visual stimuli in a pairwise comparison (PC) study with 100 Japanese (JP) stylists who have extensive coloration experience (career more than 7 years and are actively communicating on “hair transparency”), to generate quantitative transparency perception data (see Experimental section for details for standard image acquisition). The success criteria for our model would be that (i) HTI values of the swatches measured by SAMBA correlate with the ranking scores generated by the PC study, with statistically significant difference perceived by stylists among the shades captured; and (ii) a physical workshop with stylists (N = 9) to qualitatively demonstrate that the perception of these swatches presented in‐person is similar to that shown as digital images.

First, let us focus on how Japan PC study (N = 100 Japanese stylists) results matched with the HTI model. PC score was calculated as for each “win” in the pairwise comparison; the sample gets 1 point. Table 1 summarizes the PC ranking (higher score =higher transparency) generated. Cochran's Q test was performed to obtain statistically significant groups (*P* < .0001).

**TABLE 1:** Japan PC ranking score with statistical analysis. 3 bright shades (B, F, and G) are highlighted

Samples	JP PC score	JP PC win frequency (PC score/1800)	Statistical groups					
**A**	1435	0.797	1					
**B**	1293	0.718		2				
**C**	1068	0.593			3			
**D**	935	0.519			3	4		
**E**	880	0.489				4		
**F**	825	0.458				4	5	
**G**	743	0.413					5	
**H**	709	0.394					5	
**I**	581	0.323						6
**J**	531	0.295						6

**FIGURE 4:**
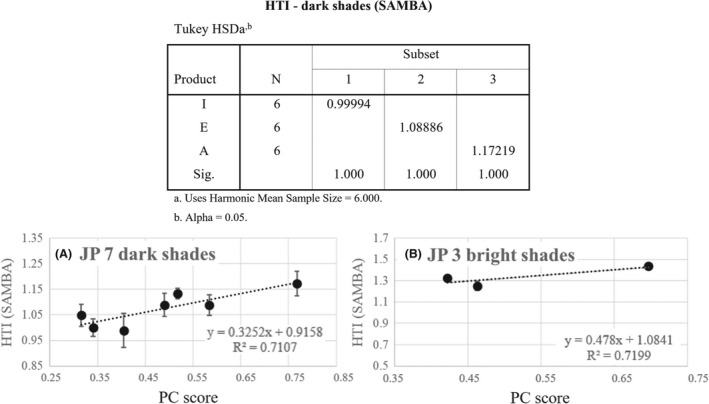
Plots of SAMBA predicted HTI values against Japan PC score (normalized by win frequency) of 10 swatches used in the study

As mentioned earlier, the comparison of HTI values of dark (${\mathrm{diffused}}_{L_{\mathrm{int}}}$ < 100) and bright groups (${\mathrm{diffused}}_{L_{\mathrm{int}}}$ > 100) is not applicable in this model, and we will discuss the results of dark group and bright group swatches separately. From Table 1, we picked 3 swatches from the dark group based on their PC scores, namely A (high transparency), E (medium transparency), and I (low transparency) and checked the HTI values of them (6 measurement points for each shade condition). As shown in the table, we were able to differentiate them in statistically significant and correct order. Figure 4A displays the relationship between HTI values and the PC scores for **dark shades**, which are linearly correlated (R^2^ = 0.71). We then conducted statistical analysis and found that Pearson correlation coefficient (PCC) = 0.843 between HTI (7 darker shades) and JP PC scores, and the correlation is significant at the 0.05 level (2‐tailed).

For the 3 bright shades, while we were able to keep the same statistical results of B/F and B/G, it seemed that there was a discrepancy between the PC results (F = G) and HTI prediction (F < G). As further increment in brightness of already bright hair color is hard to be perceived through digital images used in PC, we hypothesized that this was mainly contributed by the difference in Diffused light intensity (in SAMBA) of F (~130) and G (~200), which is perceived by SAMBA camera but not by stylists through digital images. This indicates that for bright shades (Diffused_int_ > 100) when we try to apply the HTI model to understand the impact of hair Chroma, we should select products that give similar overall Diffused intensities in SAMBA. In order to confirm this hypothesis, we chose another two bright shades (E2 and F2), which are similar in color as compared to F and G, but are of similar Diffused intensity (both are ~110) in our validation study in China (see Section 3.4).

Lastly, we confirmed the correlation between using actual swatches for transparency evaluation under standard lighting (D65 illumination) and using digital photographs by comparing the transparency score obtained in a physical workshop with 9 Japanese stylists, to the score generated by Japan PC studies. Stylists workshop (N = 9) transparency score with actual swatches (for the calculation of score, please refer to Experimental Section) showed significant correlations with Japan PC result (PCC = 0.804, at the 0.01 level). The complied results of PC scores for Japan study, scores for in‐person swatch evaluation, HTI results, and L*ab values measured by ColorShot MS (see Experimental Section for more details on ColorShot MS) of all the hair swatches used are summarized in the Appendix (Table A1).

### 3.4 Validation of the technical model with quantitative stylists’ perception data in China

Similar to the validation in Japan, there are three criteria to be met in China study (7 shades same as Japan study and 6 new additional shades from China market): (i) The 7 same shades used in both studies (in Japan and in China) have a similar and highly correlated (statistically significant) ranking results; (ii) HTI values of the swatches measured by SAMBA correlate well with the ranking scores generated by China PC study; and (iii) a physical workshop with stylists (N = 9) to qualitatively demonstrate that the perception of these swatches presented in‐person is similar to that shown as digital images.

**FIGURE 5:**
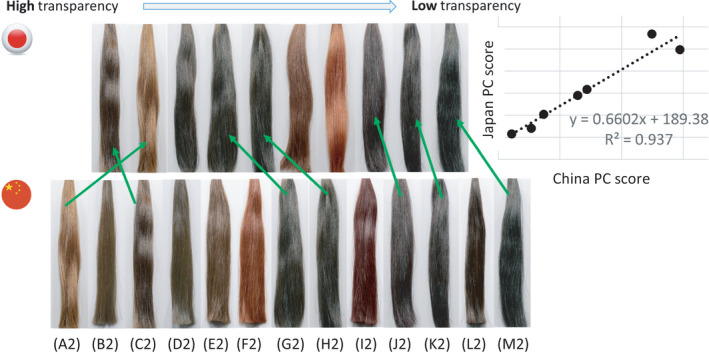
Stimuli used in Japan and China PC studies, respectively. The images are arranged in order of high transparency to low transparency rank generated by the study. Green arrows indicate the same shades used in both studies

**FIGURE 6:**
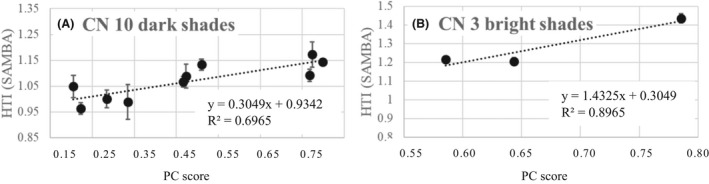
Plots of SAMBA predicted HTI values against China PC score (normalized by win frequency) of 13 swatches used in the study

The visual stimuli used are shown in Figure 5 below, and we can see that there was a good agreement in the ranking of the same shades between Japanese (JP) and Chinese (CN) stylists, and between JP and CN PC scores, Pearson correlation coefficient (PCC) = 0.968. Correlation is significant at the 0.01 level (2‐tailed). Table 2 summarizes the PC ranking (higher score =higher transparency) generated in the study in China. Cochran's Q test was performed to obtain statistically significant groups (*P* <.0001). Figure 6A displays the relationship between HTI values and the PC scores for dark shades, which fits well on a straight line. We conducted statistical analysis and found a Pearson correlation coefficient (PCC) = 0.835 between HTI and China PC scores, and the correlation is significant at the 0.01 level (2‐tailed).

**TABLE 2:** China PC ranking score with statistical analysis. 3 bright shades are highlighted in yellow

Samples	CHN PC score	Statistical groups					
**A2**	1871	1					
**B2**	1697		2				
**C2**	1647		2				
**D2**	1635		2	3			
**E2**	1587		2	3			
**F2**	1471			3			
**G2**	1120				4		
**H2**	1046				4		
**I2**	1033				4		
**J2**	769					5	
**K2**	669						6
**L2**	545						6
**M2**	510						6

For the 3 bright shades in this study, consistent with our hypothesis, with the newly selected shades (E2 and F2), we were able to achieve the same results as the PC, based on the statistical analysis, that is, A2>F2=E2. This indicates that for bright shades (Diffused_int_ > 100) when we try to apply the HTI model to understand the impact of hair Chroma, we should select products that give similar overall Diffused intensities in SAMBA.

Stylists’ workshop (N = 9) transparency score with actual swatches (for the calculation of score, please refer to Experimental Section) showed significant correlations China PC result (PCC = 0.718, at the 0.05 level).

## 4 CONCLUSION

In summary, we have demonstrated for the first time to our knowledge, the quantification of stylists’ (consumers’) hair transparency perception with objective data measured by SAMBA Hair, via a mathematical model validated with Japanese and Chinese stylists. A comprehensive study compromising of various parts was launched, starting with the qualitative identification of key parameters via in‐depth workshop discussions with over 40 stylists and a panel of 12 consumers. These parameters were then decoded quantitatively utilizing an industry standard, in‐house instruments SAMBA, which is capable of capturing the physical properties of hair fiber surface such as light reflection, diffusion, and color spectrum, enabling the numerical description of light‐hair fiber interactions for hair transparency expression. A mathematical model was then established and validated to capture the stylists’ perception of hair transparency, by two remote, online pairwise comparison studies in Japan and in China, with carefully selected anchor shades and 100 stylists in each country. Next step, we will focus on (1) expanding the scope of the study to other dark base hair rich countries, such as India and North America, and (2) building a technical performance model to link formulation science with HTI values.

## 5 EXPERIMENTAL SECTION

### 5.1 Materials

2.7g/27cm Japanese natural black hair was purchased from International Hair Importers, Inc (NY) and slightly bleached by Eiffage Energie Systèmes Game Ingénierie (alkaline solubility =10). Commercial hair coloring products from L’Oreal, Wella Professionals, and Milbon were used to color the slightly bleached hair (3 swatches per product) to achieve a wide range of color performance of the anchor shades.

### 5.2 Instruments

A SAMBA Hair System (version 3.0.2) from Bossa Nova Technologies, Venice, CA, was used to determine hair Luster (LBNT), hair Diffused profiles, to compute the Hair Transparency Index (HTI). A ColorShot system with multispectral acquisition (ColorShot MS) from Newtone Technologies was used to determine the L*, a*, b*, *C* values of hair swatches used.

### 5.3 Paired comparison study with Japanese and Chinese stylists

**FIGURE 7:**
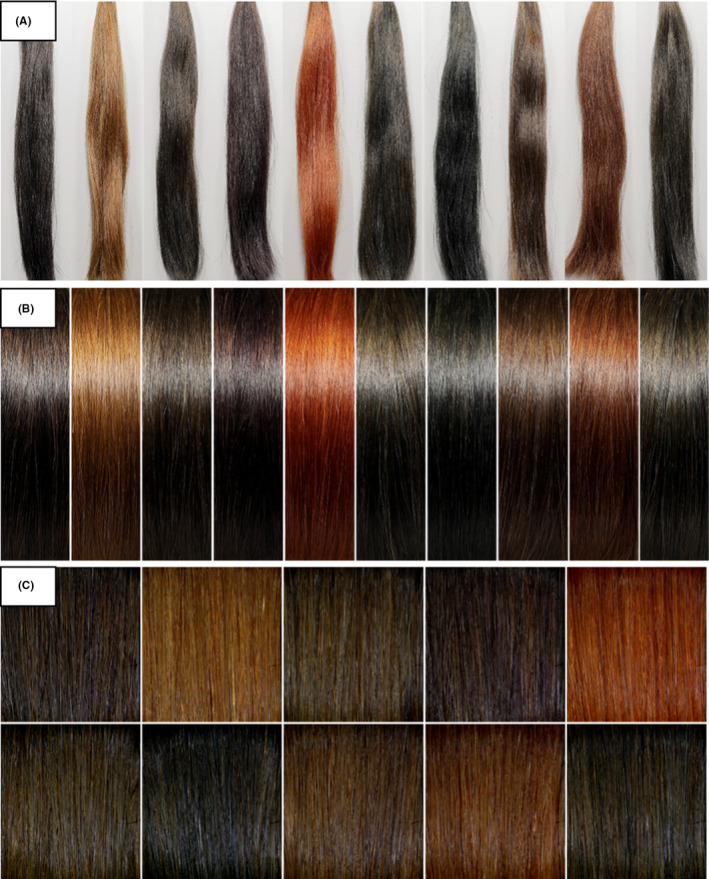
Collection of visual stimuli used in Japan PC study. Image acquisition protocol is listed in a separate section below (A) Natural look. (B) aligned and curved look. (C) images from ColorShot

*Japan*: 100 stylists with a career more than 7 years who actively communicate on hair transparency were recruited. An online platform for paired comparison study developed by Newtone Technologies was used for the study. The question asked was “*A set of pictures will be presented to you in pairs. You will have to choose the picture in which you consider to have more hair transparency by clicking on it*.” We conduct 3 separate PC studies with 3 different types of standard photographs of the 10 selected hair shades were used as visual stimuli (Natural look, aligned and curved look, and images from ColorShot, as shown in Figure 7A‐C, respectively, acquisition protocol of type (a), (b), and (c) images are discussed in the section below). We followed up the PC study with an online questionnaire, where natural look images (ie, images from 7(a)) were chosen by the stylists as the easiest to check hair transparency. So the results from this group were used for HTI model creation.

**FIGURE 8:**
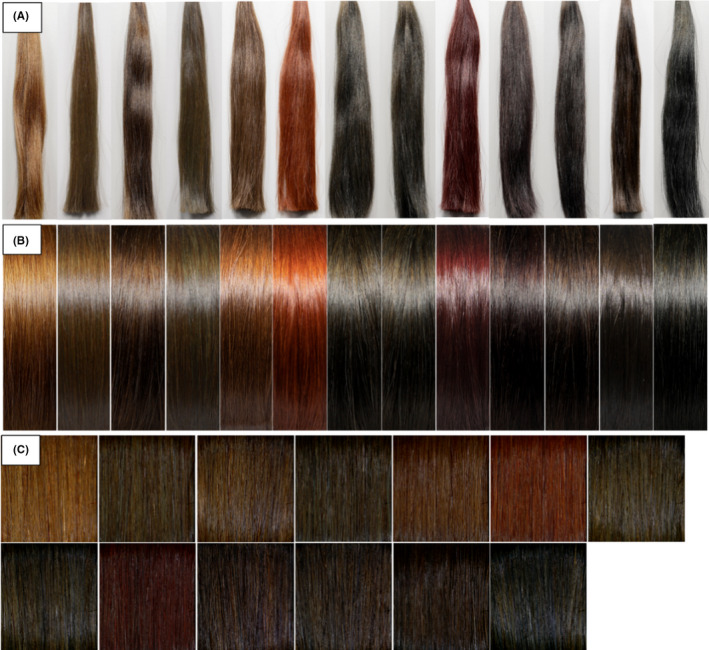
Collection of visual stimuli used in China PC study. Image acquisition protocol is listed in a separate section below (A) Natural look. (B) aligned and curved look. (C) images from ColorShot

*China*: 100 stylists with a career more than 5 years (an average of 11 years) who actively communicate on hair transparency were recruited. An online platform for paired comparison study developed by Newtone Technologies was used for the study. We conduct 3 separate PC studies with 3 different types of standard photographs of the 13 selected hair shades were used as visual stimuli (Natural look, aligned and curved look, and images from ColorShot MS, as shown in Figure 8A‐C, respectively).

After the PC study was completed, Cochran's Q test was performed to obtain statistically significant groups (*P* < .0001) by Newtone Technologies for both Japan and China studies.

### 5.4 Acquisition of standard images used in the study

**FIGURE 9:**
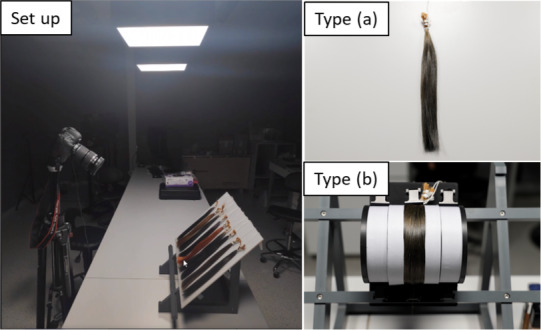
Setup of image acquisition system

Type (a) and (b) images were acquired by a digital DSLR camera (CANON EOS 6D MARK II +a lens 24‐105 IS STM) attached to a calibrated screen, illuminated under D65 light (Thouslite LED Cubes) from below (Figure 9). For type (a), hair swatches were placed directly on the acquisition whiteboard while for type (b), hair swatches were mounted to SAMBA stage to be curved and placed at the same position as type (a) for acquisition. The settings on the camera were adjusted to best match the image captured by the camera (displayed on the calibrated screen) to human‐perceived color of the swatch. Type (c) images were directly obtained from ColorShot MS manufactured by Newtone Technologies.

### 5.5 Transparency evaluation with actual swatches by Japanese stylists

9 stylists with a career more than 7 years who actively communicate on hair transparency were invited to Nihon L’Oreal Research and Innovation center for an interview, where they were presented with the hair swatches used in the PC studies under D65 lighting, and asked to group the swatches based on their level of transparency: transparent (somewhat transparent) and not transparent. The score for each swatch was calculated as such 1 point if they were in *transparent* group, 0 point in *somewhat transparent* group, and −1 point in *not transparent* group.

## CONFLICTS OF INTEREST

All authors were employees of L’OREAL at the time of the study.

## Data Availability

The data that supports the findings of this study are available in the Appendix (Table A1) of this article.

## 1

**TABLE A1:** Summary of JP stylist PC data, swatch evaluation data, HTI values, and color measurements of the swatches used

No	Shade	L*	a*	b*	PC Japan	PC China	Swatch score	HTI
1	A/C2	25.1	5.4	37.6	1435	1647	6	1.172
2	B/A2	32.5	9.5	33.7	1293	1871	4	1.435
3	C	23.6	3	42.7	1068		5	1.088
4	D/G2	23.3	1.5	47.1	935	1120	3	1.133
5	E/H2	19.5	1	41.9	880	1046	3	1.089
6	F	25.9	10.5	32.5	825		4	1.251
7	G	30	19.6	27.3	743		4	1.326
8	H/J2	18.4	4.5	42	709	769	2	0.989
9	I/K2	19.5	3.2	46.8	581	669		1
10	J/M2	17.7	0.3	54.2	531	510	2	1.049
11	B2	21.4	3.3	11.2		1697	5	1.143
12	D2	20.1	2.6	9.4		1635	3	1.092
13	E2	24	8.1	14.6		1587		1.205
14	F2	24.5	15.3	19.8		1471		1.216
15	I2	16.8	11	7.6		1033	1	1.067
16	L2	15.4	2.9	5.3		545	‐5	0.964

Note

Summary of measured data for swatches colored with different shades. SAMBA measurement data reported here are average values of 6 measurements per condition (3 swatches and 2 measurements/swatch).
